# Semi-Continuous Reverse Membrane Bioreactor in Two-Stage Anaerobic Digestion of Citrus Waste

**DOI:** 10.3390/ma11081341

**Published:** 2018-08-02

**Authors:** Tonny Kurniawan, Lukitawesa, Ilma Hanifah, Rachma Wikandari, Ria Millati, Mohammad J. Taherzadeh, Claes Niklasson

**Affiliations:** 1 Department of Food and Agricultural Product Technology, Universitas Gadjah Mada, Yogyakarta 55281, Indonesia; tonnykurniawan069@gmail.com (T.K.); ilmahanifah13@gmail.com (I.H.); rachmawikan@yahoo.com (R.W.); 2 Swedish Center for Resource Recovery, University of Borås, 50190 Borås, Sweden; lukitawesa.lukitawesa@hb.se (L.); Mohammad.taherzadeh@hb.se (M.J.T.); 3 Department of Chemistry and Chemical Engineering, Chalmers University of Technology, 41296 Gothenburg, Sweden; claesn@chalmers.se

**Keywords:** anaerobic digestion, biogas, membrane bioreactor, semi-continuous, citrus waste, two-stage

## Abstract

The presence of an antimicrobial compound called D-Limonene in citrus waste inhibits methane production from such waste in anaerobic digestion. In this work, a two-stage anaerobic digestion method is developed using reverse membrane bioreactors (rMBRs) containing cells encased in hydrophilic membranes. The purpose of encasement is to retain a high cell concentration inside the bioreactor. The effectiveness of rMBRs in reducing cell washout is evaluated. Three different system configurations, comprising rMBRs, freely suspended cells (FCs), and a combination of both (abbreviated to rMBR–FCs), are incubated at three different organic loading rates (OLRs) each, namely 0.6, 1.2, and 3.6 g COD/(L cycle). Incubation lasts for eight feeding cycles at 55 °C. Methane yield and biogas composition results show that rMBRs perform better than rMBR–FCs and FCs at all three OLRs. Volatile fatty acid profiles and H_2_ production show that the reactors are working properly and no upset occurs. Additionally, a short digestion time of 4 days can be achieved using the rMBR configuration in this study.

## 1. Introduction

Citrus, as one of the most prominent genus of fruit, has reached a global production level of 121 million metric tons per year [1]. Around 40–60% of citrus used for juice production ends up as waste [2]. This large amount of discarded waste has raised concerns about high transportation costs, lack of dumping sites, and the accumulation of organic matter. Therefore, an efficacious countermeasure is necessary to diminish the problem, and converting citrus waste into value-added products, such as biogas, is deemed attractive. Biogas is a renewable energy source that consists mainly of methane and carbon dioxide. The production of biogas involves a series of anaerobic digestion phases, and under optimal conditions, the energy output/input can reach 28 MJ/MJ, resulting in very efficient use of the biomass [3]. Due to its low cost, abundance, and high organic matter content, there are increasing attempts to utilize citrus waste as a substrate in biogas production. 

Theoretically, the high organic content in citrus waste would provide a good substrate for anaerobic digestion micro-organisms to produce biogas. However, biogas production from citrus waste poses a challenge caused by a natural inhibitor compound called D-Limonene. This chemical that is also the main essential oil in oranges (90% of the peel oil) has antimicrobial activity that hinders methane production, especially by killing the slow-growing methanogens [4]. In anaerobic digestion, methanogenesis is more sensitive to the environment than the earlier stages, due to the lack of peptidoglycan in the cell wall of methanogens [5]. Moreover, the doubling time of bacteria in hydrolysis and acidogenesis is about 1.0–1.5 days, while acetogens and methanogens have doubling times of about 1–4 and 5–15 days, respectively. Consequently, the methane-forming bacteria are more easily affected by the process conditions and require a longer retention time [6].

In the production of biogas, semi-continuous operation is gaining popularity due to its high degree of flexibility, owing to the ability to manipulate concentration levels and hence to manipulate the rate of reaction by an appropriate feeding strategy [7]. Higher biogas yield and lower technical complexity lower than batch operation make it even more favourable. The feeding system, however, promotes a high rate of cell washout at high organic loading rates (OLRs) and short holding retention times (HRTs) [8].

The conventional digestion system used for biogas production employs a single-stage system, in which all four phases of anaerobic digestion occur at the same time and place. However, it is possible to run the anaerobic digestion process with two separate vessels, working in series, each of which can be optimized for higher survival rates of the different bacterial symbioses of the two stages [3]. In the case of a substrate with a natural inhibitor, like citrus waste, the two-stage system offers another advantage by retaining part of the inhibitor in the first stage and secluding it from the second stage that contains the more sensitive methanogenic bacteria. A study by Gioannis et al. [9], which utilized food waste for biogas production, supported this notion. It was shown that through a two-stage process, where the first reactor is properly operated in order to achieve a significant net hydrogen production, a 20% higher methane yield was obtained, compared to that of a single-stage process. The higher methane production of the two-stage process was due to improved hydrolysis, acidogenesis, and acetogenesis in the first stage, which results in an increased amount of volatile fatty acids (VFA) being readily available to methanogenesis in the second stage.

To further protect the bacteria cell washout, the application of membranes is gaining positive attention owing to their ability to immobilize cells and repel specific compounds [8]. In a reverse membrane bioreactor (rMBR), cells are encapsulated in a selectively permeable membrane and therefore separated from the medium. With this configuration, it is possible to retain the cells inside the reactor and simultaneously prevent cells from coming into contact with inhibitors. In the case of hydrophobic D-Limonene, the encapsulation of cells with hydrophilic membranes is believed to offer decent protection, and thus may restore the methane yield [10,11,12]. 

In this work, the effect of membrane application in two-stage biogas production from citrus waste with semi-continuous feeding operation was evaluated. To better understand the effectiveness of membrane encasement on cell washout, the experiment was conducted with three different levels of OLRs within eight feeding cycles. Each cycle was terminated at the complete consumption of the previously introduced substrate. In addition to an evaluation of reverse membrane bioreactor performance against cell washout, optimum digestion time, and VFA consumption were also investigated.

## 2. Materials and Methods

### 2.1. Inoculum

Anaerobic digesting sludge obtained from a 3000-m^3^ thermophilic biogas plant operated at 55 °C (Sobacken, Borås, Sweden) was used as inoculum after undergoing several pre-treatment steps. Upon arrival, digesting sludge was incubated at 55 °C for 3 days to acclimatize the cells. The inoculum was then sieved through 1-mm pores and centrifuged at 10,000× *g* for 3 min to separate the supernatant and the pelleted inoculum. The latter was then used for the experiment either in the form of free cells or encapsulated with a membrane. The pelleted inoculum used had 16.1% total solids (TS) and 6.3% volatile solids (VS).

### 2.2. Substrate

Citrus wastes (with composition similar to that reported by Pourbafrani et al. [13]) were taken from Brämhult juice AB (Borås, Sweden) and acidified at 55 °C. They were anaerobically digested in a two-stage reactor (University of Borås, Borås, Sweden), consisting of an acidification reactor (the first stage) and a methanogenesis reactor (Chalmers University of Technology, Gothenburg, Sweden) (the second stage). The liquid effluent from the first stage was retrieved and used as a substrate in the second stage. Prior to feeding, it was first filtered with a rotary drum filter to remove undigested material, and the filtrate was then neutralized with 2M NaOH. During this experiment, 2 collections of the first-stage effluent were used as a substrate in the second stage, having slightly different properties as presented in Table 1. The first substrate was used for feeding Cycles 1–7, while the second substrate was used for feeding Cycle 8. Both substrates had glucose and fructose concentrations of 7.19 and 6.42 g/L, respectively. The first substrate had a COD (chemical oxygen demand) value of 20.75 g/L. As for the second substrate, it consisted of 2.5% TS (*w*/*w* % wb) and had a COD value of 31.3 g/L. The D-Limonene concentrations of both substrates were 0.2% (*w*/*v*).

### 2.3. Membrane Sachet Preparation and Cell Encasement

Cell encapsulation was prepared through the same method as described by Youngsukkasem et al. [8]. Hydrophilic poly (vinylidene fluoride) (PVDF) membranes (Durapore^®^, Thermo Fisher Scientific Inc., Gothenburg, Sweden) were shaped into identical sachets with a size of 3 × 6 cm^2^. Two sides of them were heat sealed (HPL 450 AS, Hawo, Mosbach, Germany), leaving only the upper side open for cells to be loaded. Into each membrane pocket, 3 g of pelleted inoculum was carefully injected using a 5-mL plastic syringe, and the last side was then sealed. The inoculum inside the sachets was evenly spread and the sachets were visually checked for any possible leakage. The encased cells were used immediately for biogas production. The properties of the PVDF membranes used in this work were: hydrophilic with a water flow rate of 2.5 mL/ (min cm^2^), an air flow rate of 0.15 mL/ (min cm^2^); a pore size of 0.1 µm; a thickness of 125 µm; a porosity of 70%; and a maximum operating temperature of 85 °C.

### 2.4. Bioreactor Set-Up 

**Figure 1:**
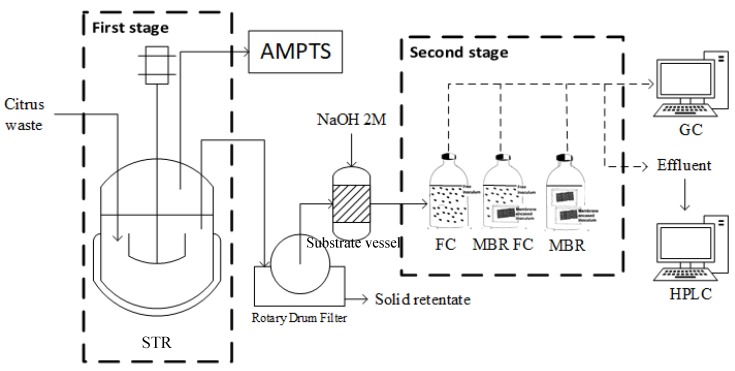
Experiment schematic diagram showing both the first and second stages. STR = stirred Tank Reactor. AMPTS = Automatic Methane Potential Test System. FCs = freely suspended cells. rMBR–FCs = combination of freely suspended cells and reverse membrane bioreactors. rMBR = reverse membrane bioreactor.

The first stage was run semi-continuously in 2-litre reactors with a working volume of 1.5 L, an HRT of 10 days, and an OLR of 5 g VS/(L day). In the second stage, the experiment was carried out in repeated batches. The inoculation was carried out using 118 mL of glass serum bottles as the reactor. Into the reactors, a fixed amount of substrate depending on the designated OLR was fed, and distilled water was added until 50 mL of a working volume was reached. Each reactor was then inoculated with 6 g of inoculum in the form according to the configuration, i.e., rMBR, FC, and a combination of both (abbreviated to rMBR–FC). For rMBR–FCs, the inoculum consisted of a mixture of 3 g of free cells and 3 g of membrane encased cells. Meanwhile, distilled water was used as a substrate for the blank. The blank was intended to quantify the gas produced by the inoculum itself, which did not derive from the consumption of substrate introduced in this experiment, thus reducing error from the external factor. In order to investigate the effect of membrane encasement on cell washout, the experiment was carried out using three different levels of OLR (0.6, 1.2, 3.6 g COD/(L cycle)). All experiments were performed in duplicate with an error of 17%. The schematic of the experiment are shown in Figure 1. 

Semi-continuous operation was carried out by feeding the reactor every time, assuming the previous substrate to be completely depleted (indicated by a cessation of methane production). To prevent extensive gas build-up that might hamper the feeding procedure and lead to vessel leakage, accumulated gas inside the reactor was first released until the pressure had reached equilibrium with the room air pressure (±1 bar). Then, the encased cells were reused, and a new substrate was introduced to the system prior to the start of every new cycle. While the reactors were being fed, the equivalent volume of effluent was also removed and kept for VFA analysis. Both feeding and effluent removal were performed using a plastic syringe with the bioreactor being placed upside-down.

### 2.5. Analysis

TS and VS of inoculum and substrate were analyzed with the thermogravimetric method, according to the laboratory analytical procedure by Sluiter et al. [14]. The COD value of the substrate was analysed using the Aquanal COD Kit (Sigma Aldrich, Riedel-de Haën, Germany). Glucose and fructose content of the substrate were determined with a Sucrose/D-Fructose/D-Glucose assay kit (Megazyme, Wicklow, Ireland).

Biogas production, yield, and composition were determined based on a measurement using Gas Chromatography (Varian 450 GC, Palo Alto, CA, USA). A gas sample (100 μL) was withdrawn using a 250-μL gas-tight syringe equipped with a pressure lock (VICI, Baton Rouge, LA, USA). It was then injected and analyzed with Varian 450 Gas Chromatography. The GC was equipped with a Wall Coated Open Tubular (WCOT, J & W Scientific GS-Gas Pro, bonded silica based 30 m × 0.32 mm, Agilent Technologies, Santa Clara, CA, USA) capillary column, a thermal conductivity detector (TCD, Varian, Palo Alto, CA, USA) and Galaxie Chromatography Data System Single Instrument as the data recording software (v.1.9, Varian, Walnut Creek, CA, USA). Nitrogen was chosen as the carrier gas with flow rates of 2.0 mL/min passing the column and of 30 mL/min passing the detector. An injection split ratio was set at 5 with an injector temperature of 75 °C. Meanwhile, the oven, detector heater, and detector filament temperatures were set at 55, 120, and 200 °C, respectively. To ascertain peak retention time and calculate gas production, pure methane (CH_4_), carbon dioxide (CO_2_), and hydrogen (H_2_) were used as the standard. Calculation was later done by comparing the peak area of the sample and the peak area of the standard, followed by conversion to the steady-state condition (273 K). Gas production was presented in accumulated gas volume under normal conditions (NmL) as a function of feeding cycle. Accordingly, gas yield was presented in gas production volume per kilogram of VS of fed substrate (Nm^3^/ kg VS) as a function of feeding cycle.

VFA content, including acetic, propionic, isobutyric, butyric, isovaleric, valeric, and caprionic acids, was analysed using High-Performance Liquid Chromatography (HPLC) (Waters, Milford, MA, USA), equipped with an ion-exchange column (Aminex HPX-87H, Biorad, Richmond, CA, USA). Autosampler (Waters) was employed for sampling. Column temperature was set at 50 °C, with a UV-VIS detector (Waters 486, Millipore, Milford, MA, USA) at 210 nm using 5 mM sulfuric acid solution (Sigma-Aldrich) as the eluent at a flow rate of 0.6 mL/min. Prior to analysis, samples were first centrifuged at 20,000× *g* for 10 min to exclude the natant from analysis. The supernatant was then filtered using an HPLC certified syringe filter (GHP Acrodisc 13 mm with 0.2 μm GHP membrane) to remove remaining contaminant particles, which might hinder column separation. The D-Limonene content in the substrate was analysed using a gas chromatography coupled to mass spectroscopy detector (GC-MS Trace GC Ultra, Themoscientific, Waltham, MA, USA) [15]. The GC equipped with silica capillary column ZB-5MS fused-silica was used to analyse a film with dimensions of 30 m × 0.25 mm × 0.25 μm, using He as the carrier gas with a flow rate of 1.2 mL/min. The temperature of the column was set to 50 °C for 2 min; it was followed by an increase of 4 °C/min up to 200 °C. The injector temperature was set at 250 °C and the detector temperature was set at 280 °C. Prior to analysis, the liquid samples were diluted with heptane in order to extract the essential oil.

## 3. Results and Discussion

In order to evaluate the performance of rMBRs in terms of their protection against cell washout in semi-continuous operation, several parameters, such as methane production and yield, biogas composition, VFA profile, and hydrogen production, were investigated. In this work, two substrates were used for the second stage, having slightly different properties as described in Section 2.2. The first substrate was fed from day-1 until day-49 (Cycles 1–7), and afterwards, it was replaced with the second substrate until day-54 (Cycle 8). The results of the experiments are as follows.

### 3.1. Methane Yield and Biogas Composition from Anaerobic Digestion of Citrus Waste

Anaerobic digestion with semi-continuous feeding under thermophilic conditions was performed with three different configurations, namely, membrane encased cells or rMBRs, FCs, and the combination of both (abbreviated to rMBR-FCs). Membrane swelling and formation of gas bubbles were observed ever since the early days of incubation, indicating that gas was being produced and released afterwards. Most membrane sachets remained unscathed throughout the process.

The new substrate was fed in repeated batches only after the previous substrate had been fully digested, indicated by a plateau on the accumulated methane curves. During the first cycle, cells were still adapting to the new environment inside the reactor, and the diffusion rate of substrates through the membranes was expected to be still quite low. This resulted in slow substrate utilization and a longer incubation period (13 days) (Table 2). On the following cycles, the time required to reach a plateau was shortened. From the time required to reach stable methane production, it can be observed that there were three distinct phases of methane production that took place: adaptation, transition, and final phase. In the first cycle, all configurations were seen to be in an adaptation phase, apparent from the long time required for all substrates to be digested. 

As incubation proceeded, D-Limonene might be appeared to accumulate inside the reactors, and cell washout from repeated feeding also started to negatively affect reactors with suspended cells. Accordingly, membrane protection against cell washout began to play an important role in methane production, and the experiment entered a transition phase. In Cycles 2 and 3, the time needed by the cells to utilize all substrate decreased (9 and 7 days were needed, respectively), emphasizing a transition to a more stable phase with shorter digestion time.

**Figure 2:**
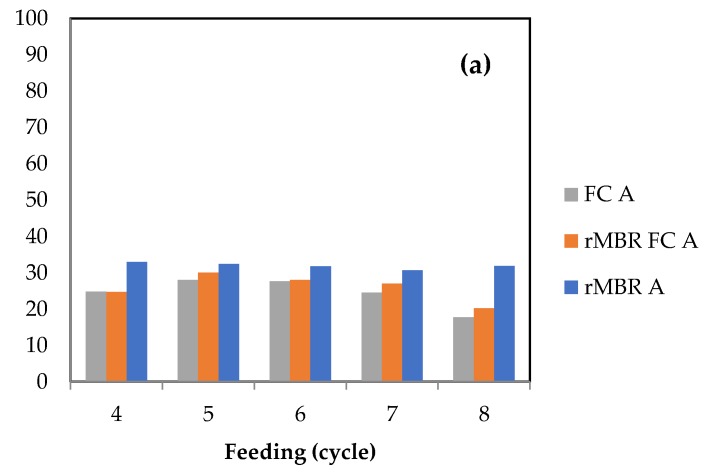

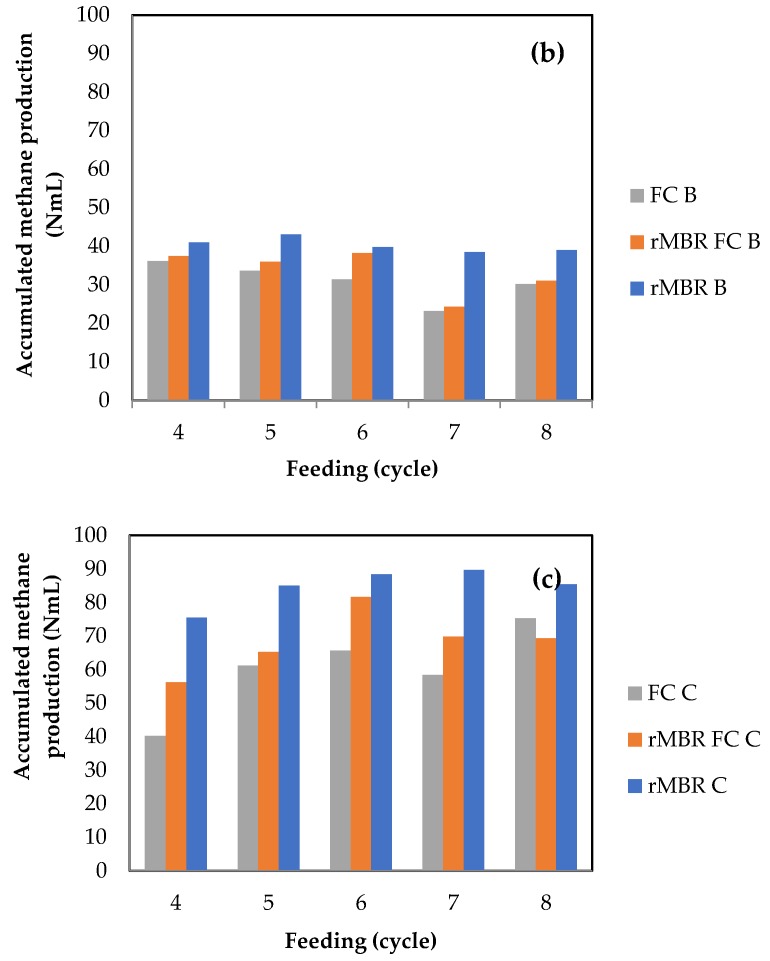
Accumulated methane production after a start-up period for OLRs: (**a**) 0.6; (**b**) 1.2; (**c**) 3.6 g VS/ (L cycle).

**Figure 3:**
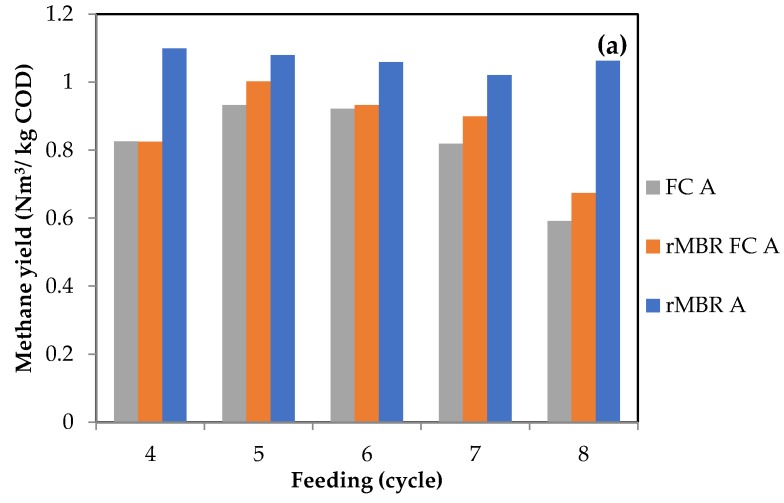

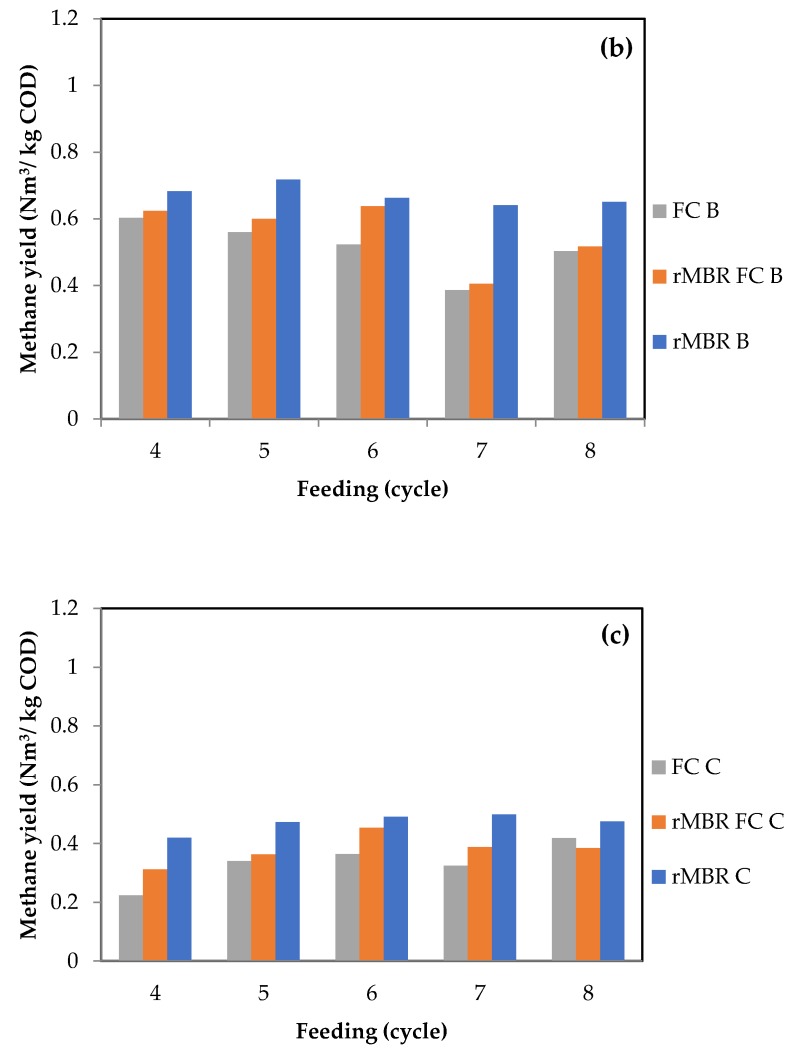
Methane yield after a start-up period for OLRs: (**a**) 0.6; (**b**) 1.2; (**c**) 3.6 g VS/(L cycle).

Starting from Cycle 4 until the last cycle, rMBRs produced the highest methane yield compared to the other configurations, which marked the final phase. Figure 2 shows accumulated methane production from the 3 different configurations in their stable phases. Encompassing the cycles altogether, the highest methane production for rMBRs, rMBR–FCs, and FCs were 33, 33, 31 (an OLR of 0.6 g COD/(L cycle)); 43, 38, 36 (an OLR of 1.2 g COD/(L cycle)); and 90, 82, 75 NmL (an OLR of 3.6 g COD/(L cycle)), respectively. The membranes’ protective effect was more pronounced at an OLR of 3.6 g COD/(L cycle) (Figure 2c) than at OLRs of 0.6 and 1.2 g COD/(L cycle) (Figure 2a,b). This was evident from the bigger volume gap between configurations. It was shown that higher OLRs resulted in higher gas production but lower methane yield (Figure 3a–c). With higher OLRs, more suspended cells were washed out; a higher amount of D-Limonene might also have entered the system and harmed suspended cells. Fully surrounded by the membranes, rMBR cells were retained inside the reactor. Previous studies using a synthetic medium and addition of different concentrations of D-Limonene showed that PVDF membranes have the ability to protect the cells from D-Limonene up to 5–10 g/L in a semi-continuous reactor [12] and 30 g/L in a batch reactor [10]. In contrast, suspended FCs in rMBR–FC and FC configurations were might be intoxicated by D-Limonene and flushed out with the effluent, reduced local cell concentration (washed out) and the methane produced.

Moreover, another outcome was discovered from this work. As observed in Figure 3, increasing the OLR from 0.6 to 3.6 g COD/(L cycle) lowered the methane yield. This meant that even though higher OLRs positively affected gas production, the same cannot be guaranteed for the yield. Hence, at higher OLRs, efficiency was reduced. More D-Limonene might permeates to the system, causing reduction in methane yield. Furthermore, no VFA accumulation (data not shown) at all OLRs applied was observed. This indicated that all reactors were working properly throughout the incubation period. 

PVDF is a semi-crystalline polymer that usually contains 59.4% fluorine and 3% hydrogen [16]. Fluoride atoms in the PVDF structure are essentially the source of membrane hydrophobicity due to the less densely packed fluorocarbons. The macromolecular linear chain structure of PVDF, which is composed of –CH_2_CF_2_– has very low surface energy. Moreover, the critical surface tensions (γc) of PVDF, –CF_2_– and –CH_2_–, which are 25 mN/m, 18mN/m, and 31mN/m, respectively, are also considered quite low. These result in a low van der Waals interaction with water, leading to poor hydrophilicity of the PVDF membrane [17]. The PVDF membranes used in this experiment were modified to possess a dominant hydrophilic property. However, the inherently hydrophobic nature of PVDF might cause some D-Limonene particles to still permeate through and access the cells, lowering the methane yield.

Another possibility is that some of the D-Limonene particles might have been smaller than the membrane pore size, allowing them to enter into the PVDF membrane. The membrane used in this experiment had a pore size of 0.1 μm. According to Grumezescu [18], D-Limonene might be present in a solution as stable nanoemulsions with particle size below 0.1 μm. This notion is further supported by a study done by Isaxon et al [19], which stated that D-Limonene particles might be shrunk into having a diameter of 95–105 nm.

Despite the considerable reduction in methane yield with higher OLRs, the result showed that rMBRs could yield up to 23% more methane than FCs. This could be caused by the role of the membranes in preventing cell washout. The average methane yield of rMBRs at an OLR of 3.6 g COD/(L cycle) in this study (0.42 Nm^3^/kg COD or equal to 0.59 Nm^3^/kg VS) was also found to be higher than the previous result (0.33 Nm^3^/kg VS) reported by Wikandari et al. [11], who applied one-stage operation with a gradient OLR up to 3 kg VS/(m^3^ day). This proved the advantage of two-stage operation. 

Moreover, in comparison with the methane yield (0.30 Nm^3^/kg VS) obtained by Millati et al. [20] who deployed a two-stage batch operation, the two-stage semi-continuous operation also exhibited an improved performance. The superiority of repeated batch operation might be due to the lower concentration of D-Limonene in the medium compared to that of batch operation.

Another interesting detail that can be extracted from this result is the determination of optimum digestion time. After Cycle 3, an incubation time of five days was set for each feeding cycle. Even so, in only 3 or 4 days, most samples had already finished producing gas. Therefore, it seems reasonable to conclude that a digestion time of 4 days was the optimum digestion time for the semi-continuous reverse membrane bioreactor in this experiment.

### 3.2. Biogas Composition

**Figure 4:**
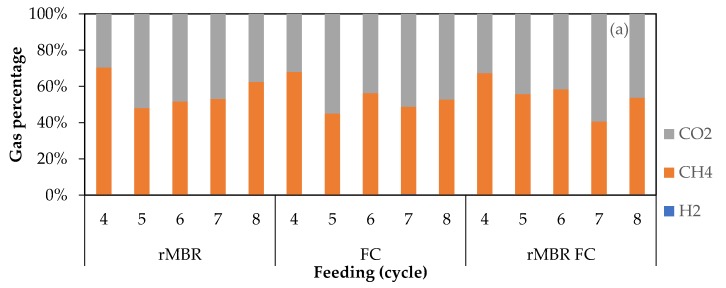

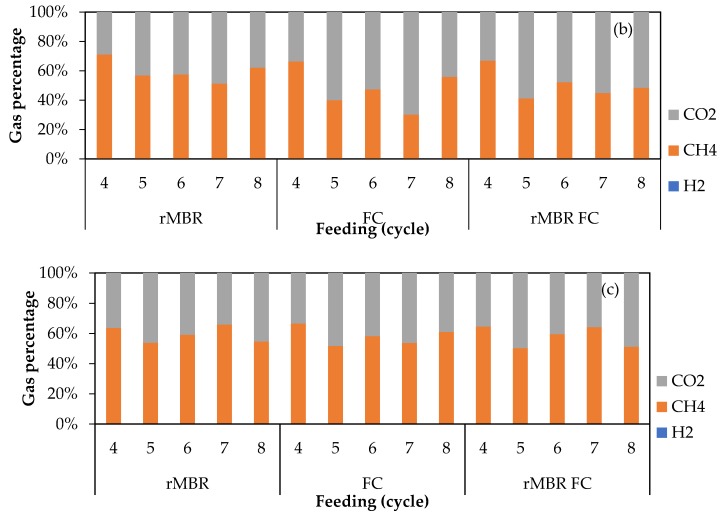
Biogas composition profile for OLRs: (**a**) 0.6; (**b**) 1.2; (**c**) 3.6 g VS/(L cycle).

In order to evaluate the effect of cell washout on biogas composition, a content ratio was determined with the assumption that only methane and carbon dioxide were produced during the digestion. From the result, the biogas composition of all samples appeared to be fluctuating with methane percentages varying within the range of 30–70%. The composition of biogas produced and the percentage range of the methane content with the different membrane configurations and OLRs are shown in Figure 4 and Table 3, respectively.

The methane content of the biogas produced from the rMBR configuration was within the reported range of 50% to 65% [3]. The methane content showed an increasing pattern as the OLR increased, but the difference was not statistically significant. Meanwhile, at the different OLRs examined, the methane content of the biogas produced from the rMBR configuration was higher than that obtained from the FC configuration. However, the hydrogen content of the biogas produced was very small (<0.9%), irrespective of the type of membrane bioreactor used. Hence, all samples could be said to be performing well throughout the incubation period based on their hydrogen production. Taking this result into account, it can be concluded that cell washout negatively affected both biogas yield and its composition when produced by a bioreactor inoculated with FCs.

## 4. Conclusions

Encasement of methane-producing bacteria in semi-permeable PVDF membranes succeeded in enhancing biogas production, utilizing inhibitor-containing citrus wastes as feedstock with semi-continuous operation at high OLRs. It was shown that reactors inoculated with membrane-encased bacteria could yield more methane than the ones inoculated with suspended cells and a combination of both configurations. Increasing the OLR, however, led to a decrease in methane yield. Nevertheless, the methane yield obtained in this research was proved to be better than that of one-stage semi-continuous operation and two-stage batch operation from previous studies.

The biogas produced by rMBRs had a higher methane percentage than that produced by FCs, showing that cell washout could be reduced by rMBR systems. Additionally, a digestion time of 4 days was found to be sufficient for the operation in this experiment.

## Figures and Tables

**Table 1:** Volatile fatty acid (VFA) content of the two effluent collections used as substrates.

	VFA Content (g/L)	
VFA	Substrate Cycles 1–7	Substrate Cycle 8
acetate	4.12	7.89
propionate	1.88	0.84
isobutyrate	0.56	0.08
butyrate	1.39	0.14
isovalerate	0.00	0.00
caproate	0.00	0.29
valerate	0.06	0.18
total VFA	8.01	9.42

**Table 2:** Required time for each configuration to reach steady (plateau) accumulated methane production.

	OLR	Time Required to Reach Steady Methane Production (Day)								
		rMBR			FC			rMBR–FC		
Feeding (cycle)		0.6	1.2	3.6	0.6	1.2	3.6	0.6	1.2	3.6
1		13	13	13	13	13	13	13	13	13
2		9	8	8	7	8	8	8	8	9
3		4	7	4	4	7	4	4	6	4
		5	4	3	3	4	3	3	5	3
5		3	3	3	3	3	3	3	3	3
6		3	4	5	3	4	4	3	5	5
7		4	5	5	4	2	4	4	3	5
8		5	5	4	4	3	4	3	4	4

**Table 3:** Range of methane percentage.

Configuration	OLR (g COD/(L Cycle))	Methane Percentage Range (%)
**rMBR**	0.6	42.7–70.4
	1.2	51.1–71.1
	3.6	53.8–73.8
**rMBR–FC**	0.6	40.5–71.6
	1.2	41.1–66.9
	3.6	50.2–68.1
**FC**	0.6	45.0–67.9
	1.2	40.0–66.3
	3.6	51.7–66.5
